# Causal conditionals and counterfactuals

**DOI:** 10.1016/j.actpsy.2012.07.001

**Published:** 2012-09

**Authors:** Caren A. Frosch, Ruth M.J. Byrne

**Affiliations:** a University of Leicester, UK; b Trinity College Dublin, University of Dublin, Ireland

**Keywords:** Conditional reasoning, Counterfactuals, Causality, Enablers, Mental models

## Abstract

Causal counterfactuals e.g., ‘if the ignition key *had been* turned then the car *would have started*’ and causal conditionals e.g., ‘if the ignition key was turned then the car started’ are understood by thinking about multiple possibilities of different sorts, as shown in six experiments using converging evidence from three different types of measures. Experiments 1a and 1b showed that conditionals that comprise enabling causes, e.g., ‘if the ignition key was turned then the car started’ primed people to read quickly conjunctions referring to the possibility of the enabler occurring without the outcome, e.g., ‘the ignition key was turned and the car did not start’. Experiments 2a and 2b showed that people paraphrased causal conditionals by using causal or temporal connectives (because, when), whereas they paraphrased causal counterfactuals by using subjunctive constructions (had…would have). Experiments 3a and 3b showed that people made different inferences from counterfactuals presented with enabling conditions compared to none. The implications of the results for alternative theories of conditionals are discussed.

## Highlights

► Causal counterfactuals understood by envisaging different sorts of possibilities. ► Causal counterfactuals are paraphrased differently compared to causal conditionals. ► People make different inferences from counterfactuals in the context of a story.

## 1 Introduction

Our primary research question is, what possibilities do people envisage when they understand a causal counterfactual, e.g., ‘if the ignition key *had been* turned the car *would have* started’? The causal counterfactual appears to convey something very different from its conditional counterpart, e.g., ‘if the ignition key was turned the car started’ (e.g., Lewis, 1973; Stalnaker, 1968). People create counterfactual alternatives to reality frequently in everyday life, when they think about how events in the past could have turned out differently, ‘if only’ (Kahneman & Tversky, 1982; Roese & Olson, 1995). The counterfactual conjecture may help them to work out the various causes of an outcome, and to prepare for the future (e.g., Mandel, Hilton, & Catellani, 2005; Markman, Klein, & Suhr, 2009). Counterfactual thoughts tend to focus on background conditions, that is, enabling causes, rather than on direct, strong causes (Byrne, 2005). For example, participants who read a story about a drunk driver who crashed into an individual driving home by an unusual route identified the cause of the accident as the drunk driver, but they created counterfactual alternatives such as ‘if only he had driven home by his usual route’ (Mandel & Lehman, 1996; N'gbala & Branscombe, 1995). They tend to focus on enabling conditions rather than strong causes, perhaps because the removal of an enabler within their control effectively prevents a bad outcome even when the cause is outside their control (Byrne, 2007; Egan, Frosch, & Hancock, 2008). And so our second question is, what possibilities do people envisage when they understand a causal conditional that refers to an enabling cause such as ‘if the ignition key was turned the car started’? The enabler is a necessary cause to bring about the outcome but it is not sufficient, that is, the outcome requires other causes to be fulfilled as well, e.g., there is petrol in the car, the battery is charged, and so on (e.g., De Neys, Schaeken, & d'Ydewalle, 2005; Markovits, Lortie Forgues, & Brunet, 2010). We report six experiments to answer these two research questions, by converging evidence from three different methods – causal conditionals as primes, paraphrases of causal conditionals and counterfactuals, and inferences from causal conditionals and counterfactuals. The experiments show that people keep in mind multiple possibilities when they think about counterfactuals, and when they think about enabling causes. First we outline how people understand and reason from ordinary conditionals, then causal conditionals, and then counterfactuals.

### 1.1 Ordinary conditionals

How do people understand and reason from conditionals? In fact, there is as yet no consensus (e.g., Byrne & Johnson-Laird, 2009). One view is that people understand an ‘ordinary’ or *indicative* conditional, ‘if there is a triangle on the blackboard then there is a circle’ (if A then B) by thinking about rules of inference, either abstract (Braine & O'Brien, 1998; Rips, 1994) or domain specific (Fiddick, Cosmides, & Tooby, 2000; Holyoak & Cheng, 1995). Another view is that they understand it by thinking about probabilities: they assume the truth of the antecedent, A, and assess whether B or not-B is more probable (Evans & Over, 2004; see also Oaksford & Chater, 2007). A third view is that they understand it by thinking about possibilities (Johnson-Laird & Byrne, 2002). A principle of truth ensures that they think about only the true possibilities that are consistent with the conditional: a triangle and a circle, no triangle and no circle, and no triangle and a circle; and they do not think about false possibilities that are ruled out by the conditional — a triangle and no circle (Espino & Byrne, 2012; Espino, Santamaria, & Byrne, 2009; Johnson-Laird & Byrne, 2002). Because of the constraints of working memory they also tend to think about few possibilities (Johnson-Laird, Byrne, & Schaeken, 1992), and so they understand the conditional by envisaging initially just a single model, a triangle and a circle (A and B), as Table 1 outlines.

On this account, people can readily make the *modus ponens* inference (A therefore B) because it matches the initial possibility they have kept in mind. They have more difficulty with the *modus tollens* inference (not-B therefore not-A) because they must think about some of the other true possibilities, e.g., not-A and not-B, in order to make it. They tend to make the *affirmation of the consequent* inference (B therefore A), whenever they keep in mind the initial possibility and fail to think of other true possibilities, e.g., not-A and B. They make the *denial of the antecedent* inference (not-A therefore not-B) when they have thought about some of the alternative possibilities (not-A and not-B) but not others (not-A and B). The interpretation of a basic conditional can be modulated by its content and context (Johnson-Laird & Byrne, 2002), as illustrated by conditionals with causal content, in the next section.

### 1.2 Causal conditionals

How do people understand and reason from *causal* conditionals? Causal conditionals can refer to different sorts of causes (e.g., Goldvarg & Johnson-Laird, 2001). They can express a strong cause, e.g., heating water to 100° causes it to boil, which is both necessary and sufficient for the outcome. They can express one of several alternative weak causes, e.g., arson caused the Australian bushfires, or accidental sparks from campfires caused them, any one of which is sufficient but not necessary. Or they can express one of several joint enabling conditions, e.g., arson caused the bushfires, enabled by the presence of dry vegetation, any one of which is necessary but not sufficient.

Alternative views exist about whether causes and enabling relations differ in terms of their meaning or logic, or in terms of characteristics such as normality, conversational relevance, constancy and covariation (e.g., Cheng & Novick, 1992; Einhorn & Hogarth, 1986; Hilton & Erb, 1996; Sloman, 2005; Turnbull & Slugoski, 1988). The interpretation of causality is controversial. One view is that people may think about different possibilities to mentally represent different sorts of causes (e.g., Frosch & Johnson-Laird, 2011; Goldvarg & Johnson-Laird, 2001; Johnson-Laird & Byrne, 1991).

Our focus is on enabling causes, and the possibilities that people consider for enabling causes. Most people consider that the enabling conditional ‘if the ignition key was turned then the car started’ is consistent with the possibility, the key was turned and the car started (A and B), and with the possibility, the key was not turned and the car did not start (not-A and not-B). But the full interpretation of the causal conditional depends on the retrieval of counterexamples (De Neys, 2011; De Neys et al., 2005; Markovits et al., 2010; see also Geiger & Oberauer, 2007). In this case people appear to think readily about disablers, e.g., the key was turned and the car did not start, perhaps because the battery was dead (A and not-B), that is, they judge the cause to be consistent with a third possibility. They do not tend to think of alternative causes, that is, possibilities consistent with the key not being turned and the car starting anyway. Their interpretation of the conditional as an enabling causal relation rules out as false the possibility that the key was not turned and the car started (not-A and B). People make different inferences from different causal relations because of the availability of counterexamples (e.g., Byrne, 1989; Byrne, Espino, & Santamaria, 1999). As a result, for an enabling cause, they make the affirmation of the consequent (B therefore A) and denial of the antecedent (not-A therefore not-B) inferences only, and they resist the modus ponens (A therefore B) and modus tollens (not-B therefore not-A) inferences, because they can retrieve a disabler — the battery being flat caused the car not to start (e.g., Cummins, Lubart, Alksnis, & Rist, 1991; De Neys et al., 2005; Markovits & Potvin, 2001).

An enabling cause can be contrasted with other sorts of causes, such as a weak cause. For example, most people judge that a cause such as ‘if the apples were ripe then they fell from the tree’ is consistent with the possibility, the apples were ripe and they fell from the tree (A and B), and with the possibility, the apples were not ripe and they did not fall from the tree (not-A and not-B). In this case people appear to think readily about counterexamples based on alternative causes, that is, they judge that the cause is consistent with a third possibility, that the apples were not ripe and they fell from the tree anyway, perhaps because of strong winds (not-A and B). They do not tend to think readily of disablers in this case, that is, possibilities consistent with the apples being ripe and not falling from the tree, and so this possibility is ruled out as false. Hence the interpretation of the conditional is as a *weak* causal relation. For a weak causal relation, they make the modus ponens (A therefore B) and modus tollens (not-B therefore not-A) inferences but they resist the affirmation of the consequent (B therefore A) and denial of the antecedent (not-A therefore not-B) inferences.

For a third sort of causal relation, a strong cause, such as ‘if Joe cut his finger then it bled’ (A causes B), people tend to think of just two possibilities: he cut his finger and it bled (A and B) and he did not cut his finger and it did not bleed (not-A and not-B), as Table 1 shows. Most people do not tend to think readily of disablers, that is, possibilities consistent with Joe cutting his finger and it not bleeding, and they do not tend to think of alternative causes, that is, possibilities consistent with Joe not cutting his finger and it bleeding — even if such possibilities exist (e.g., Cummins et al., 1991; De Neys et al., 2005). Hence they come to an interpretation of the causal relation as a strong cause, which rules out as false two possibilities: he cut his finger and it did not bleed (A and not-B) and he did not cut his finger and it bled (not-A and B). As a result, people make all four inferences from a strong cause. Enabling causes tend to be focused on when people create counterfactual conditionals, and so we turn now to a consideration of counterfactuals.

### 1.3 Counterfactual conditionals

Counterfactual conditionals often express causal claims (e.g., Thompson & Byrne, 2002), and the relation between counterfactuals and causal assertions has long been of interest to philosophers and psychologists (e.g., Byrne, 2011; Chisholm, 1946; Hoerl, McCormack, & Beck, 2011). Even with non-causal content, a counterfactual conditional in the subjunctive mood, e.g., ‘if there had been a triangle then there would have been a circle’ seems to mean something very different from an indicative one, ‘if there was a triangle then there was a circle’ (Lewis, 1973; Stalnaker, 1968). People tend to judge that someone who uttered the counterfactual meant to convey, there was not a triangle, and there was not a circle (Thompson & Byrne, 2002). When they are given an unexpected memory test after reading the counterfactual, they mistakenly recall there was not a triangle, and there was not a circle (Fillenbaum, 1974). They are primed to read quickly the conjunctions corresponding to there was not a triangle and there was not a circle when they have first read a counterfactual but not when they have read an ordinary conditional (Santamaria, Espino, & Byrne, 2005); whereas they tend to read the conjunction corresponding to there was a triangle and there was a circle equally quickly after a counterfactual and an ordinary conditional.

These results suggest that people tend to think about two possibilities when they understand a counterfactual. They think about the conjecture, a triangle and a circle, and they think about the presupposed facts, no triangle and no circle. From a counterfactual, they readily make the inferences that require access to the presupposed facts (the *modus tollens* and *denial of the antecedent* inferences) as well as the inferences that require access to the conjecture (the *modus ponens* and *affirmation of the consequent* inferences) (Byrne & Tasso, 1999; see also Egan, Garcia-Madruga, & Byrne, 2009; Moreno-Rios, Garcia-Madruga, & Byrne, 2008). They do so for counterfactuals with various sorts of content, including causal content and deontic content (Quelhas & Byrne, 2003; Thompson & Byrne, 2002).

Our aim in this paper is to examine the mental representations that people construct of causal conditionals and counterfactuals. The first two experiments (1a and 1b) examine the possibilities that are primed by indicative conditionals that express enabling causal relations. We expect that when participants read an enabling cause, e.g., ‘if the ignition key was turned then the car started’, they will readily construct not only the possibility, ‘the ignition key was turned and the car started’ but also the possibility ‘the ignition key was turned and the car did not start’ and so they will be able to read rapidly conjunctions describing these possibilities. The next two experiments (2a and 2b) compare the paraphrases that participants produce of causal conditionals and counterfactuals, e.g., ‘if the ignition key *had been* turned the car *would have* started’. We expect that their paraphrases of causal counterfactuals will reflect not only the possibility described in the counterfactual, ‘the ignition key was turned and the car started’ but also the presupposed facts ‘the ignition key was not turned and the car did not start’. The final two experiments (3a and 3b) compare the inferences people make from counterfactual conditionals when enabling conditions are made explicitly available and when they are not. We expect that when a context is provided that explicitly refers to other enabling causes, e.g., ‘there is petrol in the car’, and alternative causes, e.g. ‘the car has a start button’, inferences such as modus ponens (A therefore B) and denial of the antecedent (not-A therefore not-B) will be suppressed for counterfactuals.

## 2 Experiments 1a and 1b: enabling conditionals

The aim of Experiments 1a and 1b was to examine the sorts of possibilities people think about when they understand causal conditionals about enabling relations. We examined the possibilities that people envisage when they understand indicative causal conditionals by measuring the length of time it took them to read conjunctions (Espino et al., 2009; Santamaria, Espino, & Byrne, 2005; see also De Vega & Urrutia, 2011; De Vega, Urrutia, & Riffo, 2007; Ferguson & Sanford, 2008). Consider for example a causal conditional about a medicine bottle, ‘if the lid was twisted then the bottle opened’, presented in the context of a story in which another enabling cause has also been mentioned, ‘the lid has to be squeezed for it to open’, so that it is clear that the causal relation described in the conditional is an enabling one. When a subsequent conjunction refers to the situation in which ‘the lid was twisted but the bottle did not open’, we expect that the enabling causal conditional will prime individuals to read the conjunction rapidly, compared to a baseline control condition. We carried out two experiments to test this prediction. In Experiment 1a participants read stories that contained conditionals instantiated in 24 different contents. Experiment 1b replicated its results with a subset of 12 of these stories, presented along with filler items. The two experiments produced the same results and accordingly we report them together.

In the experiments we gave participants short stories that contained enabling relations, presented line by line on a computer screen:

**Table:** 

‘Martin was telling Laura about his medicine bottle.	Line 1
He told her that *the lid had to be squeezed for it to open*.	Line 2
He also said,	Line 3
*if the lid was twisted then the bottle opened*.	Line 4
When Martin showed Laura the bottle,	Line 5
she saw that *the lid was twisted and the bottle did not open*.	Line 6
Laura went to get a drink.’	Line 7

We presented the conditional, ‘if A then B’ (in line 4) and we ensured that it was interpreted as an enabler by presenting it in the context of an additional requirement, C which had to occur for B (in line 2). We measured the length of time it took participants to read a subsequent conjunction, in line 6 (e.g., A and not-B). In all scenarios, the target conjunction occurred in line 6 and referred to either A and B, A and not-B, not-A and B, or not-A and not-B. In Experiment 1a, we used a different content for each sort of conjunction. In Experiment 1b, we replicated the experiment using a subset of the scenarios. We again used a different content for each sort of conjunction and we ensured that the same contents were used in both the enabling and baseline conditions. We included fillers of strong and weak causes to ensure that any priming effects of enabling causes could not be attributed merely to the presence of any causal conditional.

We compared the reading times for conjunctions in the context of the extra information about the enabling causal relation, to the reading times for the same conjunctions presented in ‘baseline’ scenarios that were similar but did not contain a conditional ‘if A then B’. Instead line 4 provided filler information about A's attribute or location, e.g.,

**Table:** 

*the lid on the bottle was white*.	Line 4

We hypothesised that reading a causal conditional would prime participants to read quickly the conjunctions that describe the true possibilities that are consistent with it (Espino et al., 2009; Santamaria et al., 2005). For an enabling relation, these possibilities are A and B, not-A and not-B, A and not-B (see Table 1).

### 2.1 Method

#### 2.1.1 Participants

The participants in Experiment 1a were 22 students who participated for either course credit or 8 euro, most of whom were students at Trinity College (and 2 visiting transition year school pupils). Their mean age was 20 years (range 15–27 years) and there were 9 men and 13 women. They were selected from an initial pool of 41 volunteers based on principles for inclusion derived from Santamaria et al. (2005), namely that participants were included in the data analysis who contributed at least 83% of data points to the analysis. Data points were omitted when participants answered the questions at the end of the scenarios incorrectly or when their reading times were outliers (12 participants were excluded for answering 4 or more questions incorrectly and 9 for having a combination of 4 or more outliers and incorrect responses).

The participants in Experiment 1b were 19 volunteers recruited at the University of Reading who participated for £8. Their mean age was 30 years (range17–56 years) and there were 2 men and 17 women. They were selected from an initial pool of 34 volunteers based on the same principles for inclusion. (We therefore excluded 6 participants for answering 8 or more questions incorrectly and 9 for having a combination of 8 or more outliers and incorrect responses).

#### 2.1.2 Materials

In Experiment 1a, we employed 24 different scenarios based on the structure of the materials used by Santamaria et al. (2005). The first sentence set the scene (e.g., ‘Martin was telling his friend Laura about his medicine bottle’). The second sentence contained an additional requirement for the conditional (e.g., ‘He told her that the lid had to be squeezed for it to open’). It was designed to ensure that the relation expressed in the subsequent conditional was interpreted as an enabling condition, as its action needed to be carried out in conjunction with the action described in the conditional (e.g., squeezing the lid and twisting it). A pre-test confirmed that the materials were understood as enabling conditions. Eighteen participants, who did not take part in the main experiment, judged whether the four possible conjunctions were consistent or inconsistent with each scenario, for 38 scenarios. For the enabling conditions the conjunction A and not-B was judged as consistent 44% of the time, significantly more than for materials describing strong causes (4%) and weak causes (2%, Friedman's test, ℵ2 (2) = 22.8, *p* < .001). We selected 24 scenarios identified as the most suitable based on participants' ratings (see Appendix A).

The third sentence was of the form, ‘He also said’ and the fourth sentence was a conditional describing an enabling relation (‘If the lid was twisted then the bottle opened’). The fifth sentence was a filler (‘When Martin showed Laura the bottle, she saw that’). The sixth sentence was the target conjunction describing a possibility derived from the conditional (e.g., ‘The lid was twisted and the bottle did not open’). There were four different conjunctions, e.g., ‘the lid was twisted and the bottle opened’ (A and B), ‘the lid was twisted and the bottle did not open’ (A and not-B), ‘the lid was not twisted and the bottle opened’ (not-A and B), and ‘the lid was not twisted and the bottle did not open’ (not-A and not-B). The seventh sentence was a filler sentence about what one of the characters did next (‘Laura went to get a drink’).

The materials for the baseline condition were identical to the experimental condition, except for a change to line four of the scenario: instead of a conditional, participants were presented with a filler sentence about the colour or location of the antecedent of the conditional used in the experimental condition (‘The lid on the bottle was white’). Information about the antecedent was included in the baseline condition to ensure that the object ‘lid’ was referred to equally in the experimental and baseline conditions. All the scenarios had essentially the same syntactic structure, the same wording and the same number of words. In Experiment 1b, the materials consisted of 12 scenarios which were a subset of the materials used in Experiment 1a.

Each scenario was followed by a question about one part of the scenario. Half of those questions required a ‘yes’ response and half a ‘no’ response. We included the questions to ensure that participants were paying attention when they were reading the scenarios.

#### 2.1.3 Design

In both experiments there were two independent variables, the sort of conjunction (A and B, not-A and B, A and not-B, not-A and not-B) and the sort of scenario (enabler or baseline). The dependent measure was the reading times for the conjunctions. The design was fully within participants. In Experiment 1a there were 8 experimental conditions (2 conditions – enabler and baseline – × 4 conjunctions). Three trials of each condition were given to each participant, making a total of 24 trials, and the 24 trials were instantiated in 24 different contents. Experiment 1b had the same 8 experimental conditions (baseline or enabler condition × 4 conjunctions). Once again three trials of each condition were given to each participant, making a total of 24 trials. However, the participants received the same 12 different contents for the enabler condition (3 contents for each of the 4 conjunctions) as they did for the baseline condition. The participants also received as many filler items, based on strong and weak causes and instantiated in the same 12 contents, making a total of 48 trials.

#### 2.1.4 Procedure

The procedure was essentially the same in both experiments. We tested participants in small groups or individually. In Experiment 1a, the materials were presented on Macintosh e-Mac computers (with all extensions switched off and a CD in the CD-drive) using Superlab 1.75 software; in Experiment 1b, the experiment was presented on a PC running Windows 2000 using E-Prime software (Schneider, Eschman, & Zuccolotto, 2002). Completion of the experiment took approximately 15 min for Experiment 1a, and about 30 min for Experiment 1b. The scenarios were presented on the computer screen, one sentence at a time. Presentation of the scenarios was self-paced in that participants pressed the space bar when they were ready to move on to the next sentence. The space bar press resulted in the disappearance of the current sentence and the presentation of the next sentence. We measured how long it took them to read the conjunctions, that is, the time between the space bar press for one sentence and the following sentence.

### 2.2 Results and discussion

Based on Santamaria et al. (2005), before any data analysis we identified outliers as any latency that was less than the mean latency divided by two or greater than the mean latency plus 3 times the standard deviation. Each outlier was replaced with a missing value code and removed from the analysis. Only latencies for correct responses were analysed.

In Experiment 1a there was a main effect of condition (enabler versus baseline), *F*(1,21) = 32.48, *MSe* = .027, *p* < .001, a main effect of conjunction, *F*(3,63) = 9.45, *Mse* = .051, *p* < .001, and the two factors did not interact, *F* < 1 as shown by the 2 (conditional, baseline) by 4 (conjunction: A and B, A and not-B, not-A and B, not-A and not-B) ANOVA with repeated measures on both factors, on the log-transformed data.^1^ In Experiment 1b there was a main effect of condition (enabler, baseline); *F*(1, 18) = 9.85, *Mse* = .18, *p* = .006, a main effect of conjunction; *F*(3, 54) = 30.37, *Mse* = .04, *p* < .001, and the two factors did not interact (Greenhouse Geisser *F* = 2.23, *p* = .107).

**Fig. 1:**
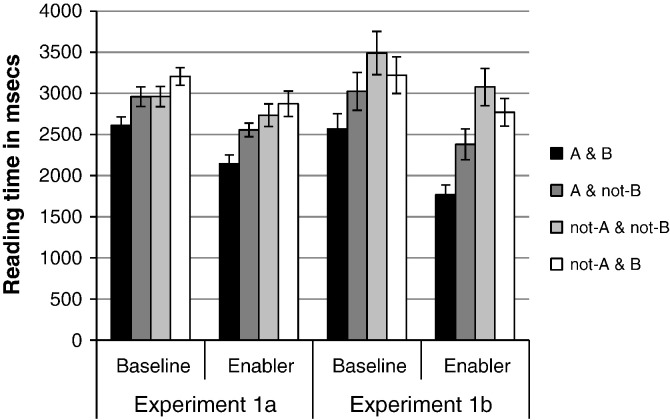
The mean reading times (in milliseconds) for the baseline and after the enabling conditional in Experiments 1a and 1b (bars are standard error).

Planned comparisons were carried out in each experiment to test our four predictions (see Winer, 1971 for a justification for carrying out planned comparisons on a non-significant interaction). As expected, the A and B conjunction was read faster when it was primed by the enabler compared to the baseline condition, in Experiment 1a *t*(21) = 4.14, *p* < .001; and Experiment 1b *t*(18) = 4.40, *p* < .001, as Fig. 1 shows. Also as expected the A and not-B conjunction was read faster when it was primed by the enabler, compared to the baseline in Experiment 1a, *t*(21) = 2.82, *p* < .01; and in Experiment 1b; *t*(18) = 2.46, *p* = .024. Unexpectedly, the not-A and not-B conjunction was not primed by the enabler, in either Experiment 1a, *t*(21) = 1.43, *p* = .166 or Experiment 1b, *t*(18) = 1.11, *p* = .281. Fourth, as expected, the not-A and B conjunction, which corresponds to the false possibility for the enabler, was not primed in either Experiment 1a; *t*(21) = 1.86, *p* = .077 or Experiment 1b; *t*(18) = 1.622, *p* = .122.

We carried out planned interaction contrasts to compare the difference between the enabler and the baseline, between each of the conjunctions. The difference between the enabler and the baseline was greater for the A and B conjunction compared to the not-A and not-B conjunction, in Experiment 1a, *F*(1, 21) = 4.418, *p* = .045, and in Experiment 1b, *F*(1, 18) = 4.319, *p* = .052. The difference between the enabler and the baseline was also greater for the A and B conjunction compared to the not-A and B conjunction, although not significantly so in Experiment 1a, *F*(1, 21) = 2.184, *p* = .151, but reliably in Experiment 1b, *F*(1, 18) = 5.449, *p* = .031. No other comparisons in Experiment 1b were significant, *F* < 1 in all cases except for the difference between the A and B conjunction and the A and not-B conjunction, *F*(1,18) = 2.517, *p* = .130.

The reliable main effect of conjunction in the two experiments reflects a trend that the A and B conjunction was read more quickly than the A and not-B one, which typically was read more quickly than the two conjunctions which start with a negation (not-A and not-B and not-A and B), as Fig. 1 shows. We did not analyse these differences as the conjunctions differ in their number of words, in particular with regard to the presence of negations, which take longer to read (Schroyens, Schaeken, & D'Ydewalle, 2001).

The A and B conjunction was primed by the enabler compared to the baseline condition, consistent with the proposal that the initial possibility that individuals think about for conditionals includes the components mentioned (Johnson-Laird & Byrne, 2002). Enablers primed two of the expected consistent possibilities A and B, and A and not-B, but not the third consistent possibility, not-A and not-B. Individuals may not have thought about this possibility because of working memory constraints (e.g., Jahn, Knauff, & Johnson-Laird, 2007). An alternative interpretation is that people interpret enablers as consistent with just these two possibilities and they consider the other possibilities to be false (i.e., not-A and B and not-A and not-B). However, such an interpretation is inconsistent with the finding from the pre-test that the not-A and not-B conjunction was rated as consistent on 89% of trials. Importantly, participants did not think about the false possibility for an enabler, not-A and B (Espino et al., 2009).

The two priming experiments provide evidence that people think about multiple possibilities when they understand causal conditionals that contain enabling causes. People envisage the possibility mentioned in the enabling causal conditional, ‘A and B’ but they also envisage the possibility ‘A and not-B’. The next two experiments examine the possibilities that people think about not only for ordinary causal conditionals but also for counterfactual causal conditionals, using a paraphrasing method. We switched to the method of paraphrasing – a deliberative interpretation task (Fillenbaum, 1976) – because our account of the differences in the mental representation of causal conditionals and counterfactuals commits us to predict that differences should be observed in deliberative interpretation.

## 3 Experiments 2a and 2b

The aim of the two experiments was to compare the mental representations of causal conditionals in the indicative mood, e.g., ‘if the ignition key was turned then the car started’ and causal counterfactuals in the subjunctive mood, ‘if the ignition key had been turned then the car would have started’. Enabling causal conditionals are understood by considering multiple possibilities, as shown in Experiments 1a and 1b. Counterfactual conditionals also require individuals to envisage multiple possibilities (Byrne, 2005). Importantly, for counterfactuals, individuals must also keep track of the epistemic status of the multiple possibilities. They must update their mental representation to reflect that one possibility corresponds to the presupposed facts and the other possibility corresponds to the conjecture. Hence we hypothesised that participants would construct different sorts of paraphrases of indicative and subjunctive causal conditionals, when asked to rephrase the conditionals without using the word ‘If’. We expected that participants would use different sorts of words to replace ‘if’ to convey the causality and counterfactuality of the conditionals. In everyday life people convey conditional relations by using a variety of connectives (Byrne & Johnson-Laird, 1992; Fillenbaum, 1974). These connectives may convey different nuances of meaning resulting in subtly different interpretations of a conditional relation (Byrne, 2007).

Causal relations can be conveyed without using ‘if’ in different ways, e.g., ‘because’, ‘so’, ‘as a consequence’, or by emphasising temporality, e.g., ‘and then’, ‘after’, ‘when’. We expected that participants would construct paraphrases of indicative causal conditionals that reflected the causally related possibilities that they thought about: for instance, we expected that participants would rely on constructions that reflect the causal (e.g., because) or temporal (e.g., when) nature of the relation and which may be considered to imply more than a single conjunctive possibility (e.g., Barrouillet & Lecas, 2000; Byrne, 2007; Snitzer Reilly, 1986). Counterfactual relations can also be conveyed without using ‘if’ in different ways, e.g., ‘should’ (e.g., ‘should A happen…’), ‘were’ (e.g, ‘were X to happen…’), or ‘had’ (e.g., ‘had X happened…’). We expected that participants would construct paraphrases of counterfactual causal conditionals that reflect not only the multiple possibilities that they have thought about, but also the nature of their epistemic status, that the conjecture differs from the presupposed facts (e.g., ‘should’, ‘had’, ‘were’). Participants were asked to paraphrase causal conditionals with enabling relations, e.g., ‘if the ignition key was turned the car started’, and also causal conditionals with strong causal relations, e.g., ‘if Joe cut his finger it bled’, and weak causal relations, e.g., ‘if the apples were ripe they fell off the tree’.

We carried out two experiments that differed only in the provision of a context for the conditional. In Experiment 2a participants paraphrased conditionals presented in isolation. Experiment 2b replicated Experiment 2a but participants were asked to paraphrase the conditionals presented in the context of explicit enablers and alternative causes to ensure that the results were independent of any differences between participants in their interpretations. The two experiments produced the same results and accordingly we report them together.

### 3.1 Method

#### 3.1.1 Participants

The participants in Experiment 2a were 30 members of Trinity College Dublin's Psychology School participant panel. There were 23 women and 7 men, whose mean age was 53 years (range 21–72 years). They received 8 euro for taking part in the experiment. One participant was eliminated from the analysis because of failure to complete the task. The participants in Experiment 2b were 41 Trinity College Dublin psychology undergraduates, who received course credit for their participation. There were 28 women and 13 men and their average age was 22 years (range 17–51 years).

#### 3.1.2 Materials

The materials for Experiment 2a were 24 conditional statements, 12 in the indicative mood and the past tense and 12 in the subjunctive mood and the past tense. In each of the 2 linguistic moods, there were 4 enabling relations, 4 strong causes, and 4 weak causes. The conditionals were adapted from Cummins (1995) and included those identified by participants there as having the following properties: (i) few alternative causes and many disabling conditions, i.e., enabling relations (e.g., if the trigger was pulled then the bullet was released), (ii) many alternative causes and few disabling conditions, i.e., weak causes (e.g., if Alvin read without his glasses then he got a headache), and (iii) few alternative causes and few disabling conditions, i.e., strong causes (e.g., if Joseph cut his finger then it bled). The materials for Experiment 2b employed the same conditionals but they were embedded in scenarios, similar to those in Experiments 1a and 1b, e.g.: ‘Jason was talking to Nancy about her car. Jason told Nancy that if the ignition key was turned, then the car started. Jason also told Nancy that the car started when the battery was charged. When Nancy went to her car she saw that the car had started. Nancy went to buy some sponges.’

In this enabling example, the scenario information ‘the car started when the battery was charged’ was designed to ensure that the causal conditional ‘if the ignition key was turned, then the car started’ was interpreted as an enabling causal relation. For a weak cause, e.g., ‘if water was poured on the campfire, then the fire went out’ the scenario information referred to an alternative cause, e.g., ‘Lisa also told Brian that the fire went out when sand was poured on it’ so that the causal conditional was interpreted as a weak cause; for a strong cause, e.g., ‘if Joseph cut his finger then it bled’ the information referred simply to the location of individuals when the consequent occurred ‘Joseph's finger bled when he was at the kitchen sink’ so that the causal conditional was interpreted as a strong cause. We included these different sorts of causes for generality; our primary interest is in the difference between causal conditionals and causal counterfactuals, and in fact there were no differences between the three sorts of causes in the paraphrases that participants produced, and so we focus on the differences between causal conditionals and causal counterfactuals.

Each participant was given 12 of these scenarios, 6 that contained an indicative conditional and 6 that contained a subjunctive conditional. Within the two blocks of 6 scenarios they received 2 strong causes, 2 weak causes and 2 enabling conditions.

#### 3.1.3 Design and procedure

In both experiments, participants acted as their own controls. The conditionals were presented in counterbalanced blocks (indicative versus subjunctive) and the types of causes were randomised within the 2 blocks. In Experiment 2a the same content was used in the indicative and subjunctive blocks and in Experiment 2b each content appeared in either the indicative mood or the subjunctive mood for different participants. There were three conjunctions and disjunctions at fixed regular intervals as fillers.

In both experiments, participants were tested individually. They were given a booklet with the following instructions, adapted from Fillenbaum (1976):

Your task will consist of rephrasing each sentence as accurately as you can. You should try to keep its meaning as much as possible, but without using the word ‘IF’. Imagine that you are rewording each sentence for someone else so that they can make sense of it as fully and exactly as possible. Your task is not to improve the sentences or make them more sensible, but to paraphrase them, rewording each in a way that captures its meaning as accurately as possible.

There was no time limit and completion of the task took about 20–30 min.

### 3.2 Results and discussion

Causal counterfactuals were paraphrased differently from causal conditionals. The results of both experiments showed that causal counterfactuals were paraphrased most often by using subjunctive constructions that preserved their counterfactuality; in contrast, indicative causal conditionals were paraphrased most often with causal or temporal connectives. Participants' paraphrases were categorised according to the type of connective that was used in place of ‘if’. Connectives were categorised based on their dictionary definitions. Five main categories of paraphrases were identified: causal, temporal, conditional, and conjunctive and a fifth category that we labelled ‘subjunctive’ (e.g., ‘had A happened B would have happened’), as shown in Table 2. Other categories had fewer than 5% of responses (e.g., disjunctive) and were not included in the analyses.

In Experiment 2a, 20% of the final overall set of paraphrases were categorised by an independent rater and there was 80% agreement on the assignments (with the exception of the subjunctive category which the independent rater had categorised as causal).^2^ A further five judges were asked to categorise 16% of the responses that had proved difficult to categorise (e.g., ‘reading without his glasses gave Alvin a headache’). In Experiment 2b an independent rater categorised 20% of the paraphrases and there was 75% agreement.

In both experiments, Wilcoxon's signed ranks tests showed that, as we expected, participants used subjunctive constructions to paraphrase counterfactual conditionals more often than indicative conditionals (Experiment 2a: 36% versus 4%, *z* = 3.72, N − Ties = 20, *p* < .001; Experiment 2b: 39% versus 13%, *z* = 3.9, N − Ties = 24, *p* < .001), as Table 3 shows. The results are consistent with our hypothesis that paraphrases of counterfactuals attempt to preserve their unique characteristic that the epistemic status of the conjecture is contrasted with the presupposed facts. In contrast, participants used temporal connectives to paraphrase indicative conditionals more often than counterfactual conditionals, as we expected (Experiment 2a: 49% versus 22%, *z* = 3.58, N − Ties = 25, *p* < .001; Experiment 2b: 53% versus 32%, Wilcoxon's *z* = 3.97, N − Ties = 34, *p* < .001), as Table 3 shows. Unexpectedly however, there were no differences between paraphrases of indicative and counterfactual conditionals that used connectives that were causal (Experiment 2a: 28% in each case; Experiment 2b: 18% versus 15%, *z* = .92, N − Ties = 21, *p* > .05 and the comparison had 80% power to detect a difference of .03). Nonetheless, the results indicate that paraphrases of indicative causal conditionals convey the multiple possibilities consistent with the causal conditional by using causal or temporal connectives. There were few paraphrases that were merely conjunctive (Experiment 2a: 9% versus 5%, *z* = 1.39, N − Ties = 11, *p* = .16, and the comparison had 80% power to detect a difference of .04; Experiment 2b: .4% in each case), and few that were conditional (Experiment 2a: 6% in each case; Experiment 2b: 10% versus 9%, *z* = .52, N − Ties = 13, *p* > .05 and the comparison had 80% power to detect a difference of .03).

Participants paraphrased counterfactual conditionals by referring not to the mentioned components (A, B) but to the presupposed facts (not-A, not-B) on 10% of trials in Experiment 2a (e.g., ‘Joseph's finger didn't bleed because he hadn't cut it’), usually in conjunction with a causal connective; they referred only to the mentioned components for indicative conditionals. In Experiment 2b, no paraphrases focused on the presupposed facts, perhaps because the context scenarios asserted that the consequent of the conditional *had* occurred (e.g., ‘Joseph's finger bled’). In both experiments, the same patterns were observed in each category for strong causes, weak causes, and enablers and there were no significant differences between them.

The results show that people paraphrased counterfactual conditionals by using subjunctive phrases such as ‘had the trigger been pulled, the bullet would have fired’. The subjunctive construction does away with the ‘if’ connective but maintains the counterfactual reference to a presupposed possibility corresponding to the facts, ‘the trigger was not pulled and the bullet did not fire’, as well as to a counterfactual conjecture, ‘the trigger was pulled and the bullet fired’. Importantly, participants did not tend to paraphrase counterfactual conditionals by using connectives such as ‘when’ or ‘because’. In contrast, they paraphrased indicative causal conditionals primarily by using causal connectives such as ‘because’ and temporal connectives such as ‘when’.

The results are consistent with the proposal that people understand an indicative causal conditional (if the ignition key was turned then the car started) by thinking initially about the causal possibilities (e.g., the ignition key was turned and the car started, the ignition key was turned and the car did not start): they can readily capture these possibilities by using temporal connectives (when the ignition key was turned the car started) or causal connectives (the car started because the ignition key was turned). The results are also consistent with the proposal that, in contrast to their understanding of a causal conditional, they understand a counterfactual conditional (if the ignition key had been turned then the car would have started) by thinking about the conjecture (the ignition key was turned and the car started), and they also think about the presupposed facts (the ignition key was not turned and the car did not start); they tend to capture these two possibilities by maintaining the subjunctive construction (had the ignition key been turned, the car would have started). Of course the data reported here do not provide evidence that participants understood the original counterfactual conditional by thinking about two possibilities; nonetheless the data are consistent, together with earlier evidence (Byrne & Tasso, 1999; Egan et al., 2009; Thompson & Byrne, 2002), with the theoretical proposal that participants understand counterfactuals by envisaging both the presupposition and the facts.

Participants' paraphrases relied on counterfactual expressions essentially equivalent to the counterfactual conditional and they clearly chose structures from their linguistic repertoire that preserved the original meaning. The use of the subjunctive construction may merely reflect a superficial strategy that provides minimal compliance with the task demands (the paraphrase removes ‘if’ but does not employ an alternative connective). However, the observation that participants in some cases refer directly to the presupposed facts in their paraphrases (not-B because not-A) suggests that their use of the subjunctive construction is a genuine attempt to capture the possibilities conveyed by the counterfactual conditional.

No paraphrases used probabilistic terms such as ‘probably’ or ‘maybe’ or ‘likely’ and very few used related terms such as ‘usually’, or ‘at times’; very few used modal auxiliaries such as ‘may’, or ‘could’ (2% of all paraphrases in Experiment 2a and 1% in Experiment 2b). On the probability view of conditionals, participants understand a causal conditional by supposing the antecedent A, assessing the likelihood of B and the likelihood of not-B, and computing a numerical figure to insert in their mental representation of the conditional to indicate their degree of belief in the causal relation, e.g. A → B 0.7 (Evans, 2007; Evans & Over, 2004; Over, Hadjichristidis, Evans, Handley, & Sloman, 2007; see also Oaksford & Chater, 2007). It seems plausible therefore to derive the prediction that individuals would attempt to capture the probabilistic degree of belief in their paraphrases. The relative absence of such terms goes against the probabilistic view.

Paraphrases of causal counterfactuals ‘if A had been then B would have been’ convey the two possibilities with which they are consistent, a possibility corresponding to the conjecture (A and B), and a possibility corresponding to the presupposed facts (not-A and not-B). The availability of these multiple possibilities has been found to increase the frequency of inferences that participants make from counterfactuals (Byrne & Tasso, 1999). In particular, participants make more of the inferences corresponding to the presupposed facts from counterfactuals compared to ordinary conditionals (not-A therefore not-B, and not-B therefore not-A). In the final two experiments, we examine the inferences that individuals make from causal counterfactuals, and we focus on causal counterfactuals presented with explicit information about other enabling causes and alternative causes. We switched to the method of measuring inferences – a reliable and long-standing indirect measure of mental representations (e.g., Johnson-Laird, 2006) – because our account of differences in the mental representations of causes and counterfactuals commits us to the prediction that there should accordingly be differences in inferences from them.

## 4 Experiment 3a and 3b

The aim of Experiments 3a and 3b was to test whether people's inferences are suppressed from a causal counterfactual when it is presented in the context of a story that makes available explicit information about other enabling causes and alternative causes, compared to when it is presented with no such information. We gave participants counterfactuals such as ‘if Jane had taken the newer drug, she would have won the race’ and compared them to ordinary conditionals ‘if Jane took the newer drug then she won the race’. Participants evaluated inferences corresponding to *modus ponens* (Jane took the newer drug therefore she won the race), *modus tollens* (Jane did not win the race therefore she did not take the newer drug), *affirmation of the consequent* (Jane won the race therefore she took the newer drug) and *denial of the antecedent* (Jane did not take the newer drug therefore she did not win the race).

In Experiment 3a we compared the inferences that participants made from (a) a causal conditional, (b) a causal counterfactual presented in isolation, and (c) a counterfactual they created themselves after they had read a story. For example, one story was about a competitive runner taking a well-known and legal painkiller with side effects of fatigue who lost a race. The story made available a conjectured causal – now counterfactual – relation (taking the newer drug causes winning the race) as well as counterexamples of disablers (taking the newer drug but still experiencing pain and not winning the race) and of alternative causes (not injuring herself, not experiencing side effects, and winning the race). We gave participants the following sort of story (adapted from Boninger, Gleicher, & Strathman, 1994, see also McCloy & Byrne, 2002):

‘Jane is a runner and since the age of eight she has competed in the sprint races in local track and field events. Up through school she had won every race in which she had competed. It was at the age of 13 that she began to dream about the Olympics. At the age of 18, before starting college, she decides to give the Olympics one all out shot. She makes the Irish Olympic team for the 400 metre race. On the day before the 400 metre race, in a freak accident during training, she sprains her left ankle. Although there is no break or fracture, when she tries to run, the pain is excruciating. Her trainer tells her about many advances in pain killing medications and assures her that she will still be able to participate. He recommends that she chooses between two drugs, both legal according to Olympic guidelines. One is a well-known painkiller that has been proved effective but also has some serious side effects including temporary nausea and drowsiness. The other painkiller is a newer and less well-known drug. Although the research suggests that the newer drug might be a more effective painkiller, its side effects are not yet known because it has not been widely used. After considerable thought, she elects to go with the more well-known drug. On the day of the race, although there is no pain in her ankle, she already begins to feel the nausea and finds herself fighting off fatigue. She finishes in fourth place, only 1 tenth of a second from a bronze medal, 4 tenths from a silver, and 5 tenths from a gold medal. After the event, she learns that some athletes in other events who were suffering from similar injuries used the other, newer drug. They felt no pain and experienced no side effects. Imagine that in the days and weeks following the race Jane thinks “if only …”. How do you think she completed this thought?

When people are asked to complete ‘if only…’ sentences, most tend to focus on the same sorts of things, such as exceptional events (Dixon & Byrne, 2011; Kahneman & Tversky, 1982), actions (Byrne & McEleney, 2000; Kahneman & Tversky, 1982), and controllable events (Girotto, Legrenzi, & Rizzo, 1991), at least when they read about the events (Pighin, Byrne, Ferrante, Gonzalez, & Girotto, 2011). Many readers complete the sentence about Jane by creating the counterfactual ‘if only Jane had taken the newer drug she would have won the race’ (Boninger et al., 1994; McCloy & Byrne, 2002). We hypothesised that reasoners would make fewer inferences from a counterfactual in the context of such a story because their interpretation of the counterfactual would be influenced by the provision of counterexamples about disablers and additional causes.

In Experiment 3b we compared the inferences they made from a counterfactual presented in isolation, to the inferences they made from a counterfactual presented to them in the context of a story without the information about disablers and additional causes, to establish that differences in inference frequency occurred because of the presence of disablers and additional causes rather than the mere presence of a story. We also compared the inferences people made from a counterfactual in a story with counterexamples, but for which they were provided with a ready made counterfactual, to establish that differences in inference frequency did not occur merely because of the self-generated nature of the counterfactual.

### 4.1 Method

#### 4.1.1 Participants

The participants in Experiment 3a were 63 volunteers from Trinity College Dublin. There were 14 men and 49 women and their age range was 15 to 49 years of age. They were assigned to one of three groups, ordinary conditional (n = 20), counterfactual (n = 21), and story-with-counterexamples (n = 22). The participants in Experiment 3b were 63 volunteers from Trinity College. There were 19 men and 43 women and their age range was 15 to 46 years of age (one participant did not disclose their age and gender). They were assigned to one of three conditions, counterfactual (n = 19), story-without-counterexamples (n = 22) and story-without-if only- (n = 22).

#### 4.1.2 Procedure

The procedure was the same in both experiments. Participants were tested in small groups. The tasks were presented in a booklet and the first page contained the following instructions: “This booklet contains three scenarios. The scenarios and associated tasks are about how people think in their daily lives and are not tests of intelligence. Each scenario is followed by a set of questions. Please read each scenario carefully and answer the questions that follow. Please answer the questions in the order in which they are presented and do not try to change your answers once you have written them.” The experiment took about 10 min.

#### 4.1.3 Materials

In Experiment 3a, the materials for the ordinary conditional condition were three conditionals in the indicative mood and the past tense: ‘if Jane took the newer drug then she won the race’, ‘if Mrs Wallace pleaded with her husband then he lived’, and ‘if the taxi driver picked up Eugene and Tina then they arrived safely’. For the counterfactual conditional condition, the three conditionals were in the subjunctive mood and the past tense, e.g., ‘if Jane had taken the newer drug then she would have won the race’. For the story-with-counterexample condition, the counterfactual inference task was presented after a story and after participants had created their own ‘if only’ counterfactual. The stories were adapted from common scenarios used in earlier studies (Boninger et al., 1994; Mandel & Lehman, 1996; Wells & Gavanski, 1989). The story asserted the facts, e.g., Jane did not take the new drug and she did not win the race, it implied a counterfactual (taking the newer drug would have led to winning the race) as well as counterexamples including potential disablers (e.g., taking the newer drug but still experiencing pain) and alternatives (e.g., not injuring herself). Participants in this condition completed a sentence about how the outcome could have turned out differently ‘if only…’.

In Experiment 3b the materials for the counterfactual condition were the same as Experiment 3a, the materials for the story-without-if-only condition were the same as Experiment 3a's story-with-counterexample condition but participants did not complete an ‘if only’ sentence completion task, and the materials for the story-without-counterexample condition were the same as Experiment 3a's story-with-counterexample condition but references to an alternative to the antecedent and outcome were removed from the stories (see Appendix A) and participants did not complete an ‘if only’ sentence completion task.

Each participant completed four sorts of inferences corresponding to *modus ponens, modus tollens, denial of the antecedent*, and *affirmation of the consequent*, for each of the three contents (12 inferences). They chose their conclusion from a set of three conclusions, e.g., (a) she won the race, (b) she did not win the race, and (c) she may or may not have won the race. Participants were scored as having endorsed an inference if they chose the option corresponding to the inference (i.e., A therefore B, B therefore A, not-A therefore not-B, not-B therefore not-A). For example, for the *modus ponens* premises, ‘if Jane took the newer drug then she won the race. Jane took the newer drug’, participants were scored as having endorsed the inference if they chose option (a) above, and as not having endorsed it if they chose (b) or (c). The four inferences were presented in a randomised order for each participant within each block corresponding to each content, and the three contents were presented in six counterbalanced orders.

### 4.2 Results and discussion

In Experiment 3a, Mann Whitney tests on the comparison between ordinary conditionals and counterfactuals showed that participants made more *modus tollens* inferences from counterfactuals (81%^3^ versus 58%, *U* = 142.5, n1 = 21, n2 = 20, *z* = − 1.934, *p* = .053); the difference for the *denial of the antecedent* inference was not significant (46% versus 20%, *U* = 144.5, *z* = − 1.828, *p* = .068), as Table 4 shows. They made the same frequency of *modus ponens* (86% versus 80% *U* = 195, *z* = −.476, *p* = .63) and *affirmation of the consequent* inferences (46% versus 40% *U* = 190, *z* = −.543, *p* = .59) in the two conditions. These results replicate earlier studies (Byrne & Tasso, 1999; Egan et al., 2009; Thompson & Byrne, 2002).

As we predicted, participants made fewer inferences from the counterfactual presented in a story with counterexamples compared to the counterfactual presented in isolation, for *modus ponens* (49% versus 86%, *U* = 102, n1 = 22, n2 = 21, *z* = − 3.338, *p* = .001), and *modus tollens* inferences (45% versus 81%, *U* = 118, *z* = − 2.924, *p* = .003). There were no differences for the *affirmation of the consequent* (53% versus 46%, *U* = 204, *z* = −.691, *p* = .49) and *denial of the antecedent* inferences (59% versus 46% %, *U* = 190, *z* = − 1.045, *p* = .296).

For the story-with-counterexamples, participants completed an ‘if only…’ sentence. Their ‘if only’ sentences corresponded to the expected counterfactual, e.g., ‘if she had taken the other drug…’ on 67% of trials; their remaining ‘if only’ thoughts tended to focus on a prior event that had led up to the counterfactual choice, e.g., ‘if only she hadn't injured her ankle…’. The ‘if only’ thoughts generated by participants validate the idea that the majority of participants produced the same counterfactual thought as the one participants were asked to evaluate in the inference task. Therefore, the conditional they were reasoning from was consistent with their beliefs about the facts of the story. It also confirms that they accepted the counterfactual alternative suggested by the story as a way in which the outcome could have been different.

In Experiment 3b, participants also made fewer inferences from a counterfactual presented in a story with counterexamples but without the requirement to generate the ‘if only’ counterfactual, compared to the counterfactual presented in isolation, for *modus ponens* (42% versus 86%, *U* = 79, n1 = 19, n2 = 22, *z* = − 3.597, *p* = .001), *modus tollens* inferences (48% versus 81%, *U* = 110, *z* = − 2.735, *p* = .006), *affirmation of the consequent* (39% versus 63%, *U* = 136, *z* = − 1.988, *p* = .047) and *denial of the antecedent* (32% versus 58%, *U* = 125, *z* = − 2.305, *p* = .021). The result confirms that it is the presence of counterexamples in the story that is crucial rather than merely the requirement to generate the ‘if only’ counterfactual.

There were no significant differences between the inferences they made from the counterfactual presented in the context of a story with no counterexamples and a counterfactual presented in isolation, for *modus ponens* (86% versus 68%, *U* = 147, n1 = 19, n2 = 22, *z* = − 1.827, *p* = .068), *modus tollens* (81% versus 59%, *U* = 142, *z* = − 1.897, *p* = .058), *affirmation of the consequent* (63% versus 44%, *U* = 146, *z* = − 1.704, *p* = .088) and *denial of the antecedent* (58% versus 41%, *U* = 161, *z* = − 1.318, *p* = .188). This null result is suggestive that the presence of counterexamples mediates the suppression of the inferences, rather than merely the presence of a story.

Jonckheere's trend tests on the results show a reliable trend in the inferences from the counterfactual in isolation, the counterfactual in the context of a story-without-counterexamples, and the counterfactual in the context of a story-without-if-only, for *modus ponens* (86%, 68% and 42%, *J* = 942, N = 63, *p* = .001), *modus tollens* (81%, 59%, 48%, *J* = 865, *p* = .007), *affirmation of the consequent* (63%, 44%, 39%, *J* = 815, *p* = .043) and the *denial of the antecedent* inferences (58%, 41%, 32%, *J* = 819, *p* = .036).

The results of Experiment 3a show that reasoners made fewer *modus ponens* and *tollens* inferences when they read a story that provided counterexamples and that required them to create the ‘if only’ counterfactual. The results of Experiment 3b showed that they made fewer of all four inferences when they read a story that provided counterexamples even when it did not require them to create the ‘if only’ counterfactual. The effect may have been observed more clearly in Experiment 3b because the removal of the requirement to create the counterfactual may have removed the variability caused by some individuals generating different counterfactuals from the target one.

## 5 General discussion

How do people understand and reason from causal counterfactuals, e.g., ‘if the battery *had been* charged the car *would have* started’ compared to causal conditionals, e.g., ‘if the battery was charged then the car started’? In particular what possibilities do people envisage when they understand a causal counterfactual, and a causal conditional that refers to an enabling cause? The six experiments we have reported to address these research questions provide converging evidence from three different methods — causal conditionals as primes, paraphrases of causal conditionals and counterfactuals, and inferences from causal conditionals and counterfactuals. The results show that (a) people are primed by an enabling causal conditional to read more quickly conjunctions corresponding to the possibilities ‘the battery was charged and the car started’ and ‘the battery was charged and the car did not start’, (b) people produce paraphrases for causal counterfactuals that capture their counterfactuality (e.g., would…, had…, were…) whereas they produce paraphrases for causal conditionals that emphasise their temporal-causality (e.g., after, and so), and (c) people make fewer inferences from causal counterfactuals presented in a story with counterexamples, compared to a causal counterfactual in isolation, or a causal counterfactual in a story with no counterexamples. We consider the implications of these three findings in turn.

First, the first two experiments (1a and 1b) examined the latencies to read conjunctions corresponding to A and B, A and not-B, not-A and B, and Not-A and Not-B, after participants had first read an enabling causal conditional (if A then B). The results showed that enabling causal conditionals primed two of their consistent possibilities, A and B, and A and not-B. The data provide support for the idea that people think initially about two consistent possibilities when they understand the cause (see also Sloman, Barbey, & Hotaling, 2009; Wolff, 2007). The priming results are inconsistent with the idea that individuals evaluate their belief in a conditional by adding the antecedent to their beliefs and assessing whether the consequent does or does not occur, as probabilistic theories propose, that is, that they think about A and B and A and not-B (Evans & Over, 2004; Over et al., 2007, see also Oaksford & Chater, 2007). Likewise, the data are incompatible with the view that people evaluate a causal relation by making a contingency judgement based on assessing the cases in which the effect is present, with or without the cause, that is, that they think about A and B and not-A and B (Cheng & Nisbett, 1993).

Second, the mental representation of causal indicative and subjunctive conditionals is different. The second two experiments (2a and 2b) examined the paraphrases that participants produce of indicative and counterfactual conditionals that express different causal relations. They showed that people create different paraphrases for causal conditionals in the indicative and the subjunctive moods. People paraphrase indicative causal conditionals without using ‘if’ by using causal or temporal connectives (e.g., because, when); they paraphrase counterfactual causal conditionals by using subjunctive constructions (e.g., had A happened, B would have happened). We suggest that the different paraphrases that individuals construct reflect their different mental representations (Fillenbaum, 1974; Johnson-Laird & Byrne, 1992). They rely on temporal and causal connectives to capture the causal possibilities that they have thought about initially (e.g., for enablers: A and B, A and not-B) when they understand an ordinary causal conditional (if A then B). In contrast, they often rely on subjunctive constructions to capture the epistemically different possibilities corresponding to the conjecture and the presupposed facts (A and B, not-A and not-B) when they understand a counterfactual conditional (if A had happened, B would have happened). The subjunctive paraphrase (had A happened B would have happened) may reflect a superficial linguistic strategy rather than the possibilities individuals have kept in mind (e.g., Ormerod, Manktelow, & Jones, 1993). Nonetheless, the reference to the presupposed facts (not-A and not-B) in some of the paraphrases suggests that the subjunctive construction is a genuine attempt to capture the possibilities conveyed by the counterfactual conditional.

Third, people make different inferences from causal counterfactuals that they interpret in a story with counterexamples, compared to those interpreted in isolation. The final two experiments (3a and 3b) examined the inferences people make from causal counterfactuals interpreted in isolation compared to those interpreted in a story with counterexamples. People make fewer inferences from counterfactual conditionals in the context of a story with disablers and alternative causes compared to counterfactuals in isolation or counterfactuals in a story with no counterexamples.

The experiments provide converging evidence from three very different methods, paraphrases, priming, and inferences, that causal conditionals and causal counterfactuals are understood by thinking about possibilities of different sorts. Converging evidence from different tasks may be helpful in developing a full understanding of the way in which people mentally represent causal conditionals.

## Figures and Tables

**Table 1:** The consistent possibilities for indicative and counterfactual conditionals expressing basic content and enabling causal relations; with information on strong and weak causes for comparison.

	Indicative	Counterfactual	
	*If A then B*	*If A had been then B would have been*	
Basic	A and B	A and B	(Conjecture)
	Not-A and not-B	Not-A and not-B	(Presupposed facts)
	Not-A and B	Not-A and B	
Enabler	A and B	A and B	(Conjecture)
	Not-A and not-B	Not-A and not-B	(Presupposed facts)
	A and not-B	A and not-B	
Strong	A and B	A and B	(Conjecture)
Cause	Not-A and not-B	Not-A and not-B	(Presupposed facts)
Weak	A and B	A and B	(Conjecture)
Cause	Not-A and not-B	Not-A and not-B	(Presupposed facts)
	Not-A and B	Not-A and B	

**Table 2:** Categories of connectives used in the paraphrases produced in Experiments 2a and 2b.

Category	Connectives
Temporal	When, and then, then, after, as soon as, whenever, following, once, upon, on, always B each time A
Causal	By, causes, because, in order, as, so, for, indicates, shows, as a result of, as a consequence, usually produces, A-ing…B
Conditional	Provided, to, means, in the event of
Subjunctive	Should…would (i.e., ‘should Joseph cut his finger, it would bleed’) Had…. would have (e.g., ‘had the match been struck, the flame would have appeared’), would have …had Were …would (e.g., ‘were Alvin to read without his glasses, he would get a headache’)
Conjunctive	And, and therefore

Note: some connectives may be considered to belong to more than one category, e.g., ‘as a result of’ can be considered to be both temporal and causal; in these cases we assigned the connective to the category on the basis of its primary use in the paraphrase.

**Table 3:** Percentages of each type of connective as a function of type of conditional, indicative or subjunctive in Experiments 2a and 2b.

Connective	Indicative		Subjunctive	
Experiment	2a	2b	2a	2b
Temporal	49	53	22	32
Causal	28	18	28	15
Subjunctive	4	13	36	39
Conditional	6	10	6	9
Conjunctive	9	0.4	5	0.4

Note: only categories with 5% or more responses in one cell were included.

**Table 4:** Percentages of each type of inference endorsed in the conditions of Experiment 3a and 3b (the remainder in each cell is the percentage of responses of the opposite of the inference or responses that the conclusion may or may not follow).

Inference	MP	AC	MT	DA
Experiment 3a				
Conditional	80	40	58	20
Counterfactual	86	46	81	46
Story with counterexamples	49	53	45	59

Experiment 3b				
Counterfactual	86	63	81	58
Story with counterexamples and without ‘if only’	42	39	48	32
Story without counterexamples	68	44	59	41

Key: MP = modus ponens, AC = affirmation of the consequent, MT = modus tollens, and DA = denial of the antecedent.

## Appendix A

1. Conditionals for Experiment 1a and 1b presented with the additional enabler (conditionals marked with * were used in Experiment 1b).
*If the key was turned, then the car started.
  He told her that the battery had to be charged for the car to start.
*If the button was pushed, then the bell rang.
  He told her that the wires had to be connected for the bell to ring.
*If the lever was pulled, then the chair descended.
  She told him that a person had to be seated for the chair to descend.
*If the soil was watered, then the plants grew.
  She told him that the sun had to shine for the plants to grow.
*If the sun was shining, then the tan appeared.
  She told him that the lotion had to be used for the tan to appear.
*If his hands were bare, then his fingerprints marked.
  He told her that the object had to be touched for his fingerprints to mark.
*If the bowl was covered, then the dough doubled.
  She told him that the mixture had to contain yeast for the dough to double.
*If the lid was twisted, then the bottle opened.
  He told her that the sides had to be squeezed for the bottle to open.
*If the button was pushed, then the gadget launched.
  He told her that the object had to be vertical for the gadget to launch.
*If the coal was used, then the BBQ lit.
  He told her that the fire-lighter had to be set for the BBQ to light.
*If the inside was touched then the pollen stained.
  He told her that the buds had to be open for the lilies to stain.
*If the windows were closed, then the room cooled.
  She told him that the conditioning had to be on for the room to cool.
If the mixture was heated, then the cake rose.
  She told him that the baking-powder had to be added for the cake to rise.
If the trigger was pulled, then the gun fired.
  He told him that the bullets had to be inserted for the gun to fire.
If the needle was threaded, then the machine worked.
  She told her that the pedal had to be pressed for the machine to work.
If the switch was flipped, then the light shone.
  She told him that the bulb had to be inserted for the light to shine.
If the temperature was below freezing, then the snow fell.
  She told him that the air had to be moist for the snow to fall.
If the tap was turned, then the shower operated.
  She told him that the cord had to be pulled for the shower to operate.
If the batteries were charged, then the remote worked.
  She told him that the button had to be pressed for the remote to work.
If the plug was connected, then the headphones worked.
  He told her that the cable had to be intact for the headphones to work.
If the chemicals were combined, then the mixture darkened.
  She told him that the beakers had to be heated for the mixture to darken.
If the book was fiction, then her grandmother read.
  She told him that the print had to be large for her grandmother to read.
If the calculator was permitted, then the student succeeded.
  She told him that the tables had to be used for the students to succeed.
If the brake was depressed, then the car slowed.
  She told him a lower gear had to be used for the car to slow.
2. Conditionals for Experiments 2a and 2b in italics, presented in the context of a story in Experiment 2b only (counterfactuals were the same conditionals in the subjunctive mood).
  *Enabling conditions*
    Marcus was talking to Steven about his new gun. Marcus told Steven that *If the trigger was pulled, then the gun fired*. Marcus also told Steven that the gun fired when a bullet was in the chamber. When Steven saw the gun he saw that The gun had fired. Steven went to watch a shooting competition.
    Katy was talking to Jimmy about the kitchen light. Katy told Jimmy that *If the switch was flipped up, then the light went on*. Katy also told Jimmy that the light went on when a bulb was in the fitting. When Jimmy walked into the kitchen he saw that The light had gone on. Jimmy went to the supermarket.
    Jason was talking to Nancy about her car. Jason told Nancy that *If the ignition key was turned, then the car started*. Jason also told Nancy that the car started when the battery was charged. When Nancy went to her car she saw that The car had started. Nancy went to buy some sponges.
    Curtis was talking to Sarah about matches. Curtis said that *If the match was struck, then it lit*. Curtis also said that the match lit when the match was dry. When Sarah went into the living room she saw that The match was lit. Sarah went to get some cookies.
  *Strong causes*
    Peter was talking to Mary about his friend Joseph. Peter told Mary that *If Joseph cut his finger, then it bled*. Peter also told Mary that Joseph stood by the kitchen sink when he bled. When Mary went into the kitchen she saw that Joseph was bleeding. Mary went to welcome the new guests.
    Andrew was talking to Jenny about a murder suspect Larry. Andrew told Jenny that *If Larry grasped the glass with his bare hands, then his fingerprints were on it*. Andrew also told Jenny that Larry was in the living room when he left his fingerprints. When the forensic report arrived Jenny saw that Larry's fingerprints were on the glass. Jenny went to the canteen.
    Julie was talking to Suzanne about her boss' house in the suburbs. Julie told Suzanne that *If the doorbell was pushed, then it rang*. Julie also told Suzanne that her boss was in the garden when the doorbell rang. When Suzanne arrived at the house she saw that The doorbell rang. Suzanne went into the living room.
    Lisa was talking to David about a Buddhist temple. Lisa told David that *If the gong was struck, then it sounded*. Lisa also told David that the monks were by the pond when the gong sounded. When David went into the temple he saw that The gong sounded. David went to a meditation class.
  *Weak causes*
    Martin was talking to Thomas about the apples in the garden. Martin told Thomas that *If the apples were ripe, then they fell from the tree*. Martin also told Thomas that the apples fell from the tree when it was stormy. When Thomas went into the garden he saw that The apples had fallen from the tree. Thomas went to pick some flowers.
    Andy was talking to Tara about his friend Mary. Andy told Tara that *If Mary jumped into the swimming pool, then she got wet*. Andy also told Tara that Mary got wet when she had a shower. When Tara saw Mary she saw that Mary was wet. Tara went for a massage.
    Lisa was talking to Brian about the campfire. Lisa told Brian that *If water was poured on the campfire, then the fire went out*. Lisa also told Brian that the fire went out when sand was poured on it. When Brian went to look at the campfire he saw that The fire had gone out. Brian went to brush his teeth.
    Rita was talking to Sinead about her son Alvin. Rita told Sinead that *If Alvin read without his glasses, then he got a headache*. Rita also told Sinead that Alvin got a headache when he ate chocolate. When Sinead saw Alvin she saw that Alvin had got a headache. Sinead went to an art exhibition.
3. Stories for Experiment 3a and 3b (counterexamples information in italics)

*Runner story*
  Jane is a runner and since the age of eight she has competed in the sprint races in local track and field events. Up through school she had won every race in which she had competed. It was at the age of 13 that she began to dream about the Olympics. At the age of 18, before starting college, she decides to give the Olympics one all out shot. She makes the Irish Olympic team for the 400 metre race. On the day before the 400 metre race, in a freak accident during training, she sprains her left ankle. Although there is no break or fracture, when she tries to run, the pain is excruciating. Her trainer tells her about many advances in pain killing medications and assures her that she will still be able to participate. He recommends that she takes a drug, legal according to Olympic guidelines. It is a well-known painkiller that has been proved effective but also has some serious side effects including temporary nausea and drowsiness. On the day of the race, although there is no pain in her ankle, she already begins to feel the nausea and finds herself fighting off fatigue. She finishes in fourth place, only 1 tenth of a second from a bronze medal, 4 tenths from a silver, and 5 tenths from a gold medal. *He recommends that she chooses between two drugs, both legal according to Olympic guidelines. One is a well-known painkiller that has been proved effective but also has some serious side effects including temporary nausea and drowsiness. The other painkiller is a newer and less well-known drug. Although the research suggests that the newer drug might be a more effective painkiller, its side effects are not yet known because it has not been widely used. After considerable thought, she elects to go with the more well-known drug. After the event, she learns that some athletes in other events who were suffering from similar injuries used the other, newer drug. They felt no pain and experienced no side effects*.
*Plane story*
  Mrs. Wallace was somewhat distressed about her husband flying from Dublin to London for a convention. She herself was afraid of flying, and neither her nor her husband had ever flown anywhere before. Mr. Wallace, although a little uneasy about flying for the first time, tried to assure his wife that everything would be alright. These assurances did little to put her at ease. One week later, Mr. Wallace took his flight to London. About midway through the flight, the plane crashed, tragically killing Mr. Wallace and all others on board. In the weeks following the crash, a formal investigation found evidence from the debris and flight recorder that indicated that the plane's engine had spontaneously malfunctioned, leaving the pilot no time for an emergency landing. The investigation also determined that the plane's engine was inspected thoroughly by a qualified maintenance team prior to takeoff. Other plane engines of the same model were also inspected for structural flaws in manufacturing, but this inspection revealed that the engines were well constructed. *In the past, they had either taken the fast ferry and driven or taken the train to their destination. On this occasion, however, Mrs. Wallace was not accompanying her husband on this two day business trip. Mr. Wallace told his wife that he was going to book a flight because he didn't want to spend so many hours on the ferry and driving or taking the train. Mr. Wallace booked a flight even though he had originally considered taking the ferry. She thought about pleading with him to take the train instead. But she didn't because she felt silly doing so, even though she knew that her husband would definitely have changed his plans at her request if she pleaded.*
Taxi story
  Eugene and Tina were a young married couple who lived in the country. Both were partially paralysed and confined to wheelchairs. They had met four years before when Tina was a counsellor with the Irish Paraplegic Association, had fallen in love, and were married one year later. On this particular evening, they were going into town. Eugene and Tina took Tina's car, which was equipped with special hand controls. In order to get into town from their house, they had to travel across a bridge over Rupert River. A severe storm the night before had weakened the structure of the bridge. About 5 min before Eugene and Tina reached it, a section of the bridge collapsed. In the dark, Eugene and Tina drove off the collapsed bridge and plummeted into the river below. Both of them were badly injured. *On this particular evening, Eugene had phoned to request a taxi to take them into town. When the taxi driver arrived, Eugene and Tina were waiting by the street. On seeing that they were both in wheelchairs, the taxi driver refused their fare because he thought it would be too crowded in the taxi with both of them and the wheelchairs. So the taxi driver headed back into town without them. Because there was no time to call another taxi, Eugene and Tina took Tina's car, which was equipped with special hand controls. In order to get into town from their house, they had to travel across a bridge over Rupert River. A severe storm the night before had weakened the structure of the bridge. About 5 minutes before Eugene and Tina reached it, a section of the bridge collapsed. In the dark, Eugene and Tina drove off the collapsed bridge and plummeted into the river below. Both of them were badly injured. It is later reported that the taxi driver had reached the bridge about 15 minutes before them, and made it safely across*.

## Footnotes

1 In Experiment 1a, consistent with our inclusion criteria described above, 51 of 528 reaction times (10%) were not included in the analysis, 41 due to incorrect responses and 10 due to outliers. In Experiment 1b 100 of 912 reaction times (11%) were not included in the analysis, 70 due to incorrect responses and 30 due to outliers.

2 Of the 696 responses (29 participants × 24 paraphrases), 3% were eliminated because they used ‘if’ in the paraphrase contrary to the instructions, or merely asserted ‘yes’, or provided an explanation rather than a paraphrase.

3 The percentage reflects the fact that the *modus tollens* inference was endorsed on 81% of trials (i.e. participants given if A then B, not-B, selected the option corresponding to not-A). On the remaining 19% of trials, the opposite of the inference was endorsed (the option corresponding to A), or the indeterminate option was chosen (the option corresponding to A may or may not occur).
